# Foamability of Cellulose Palmitate Using Various Physical Blowing Agents in the Extrusion Process

**DOI:** 10.3390/polym13152416

**Published:** 2021-07-23

**Authors:** Teijo Rokkonen, Pia Willberg-Keyriläinen, Jarmo Ropponen, Tero Malm

**Affiliations:** 1 VTT Technical Research Centre of Finland Ltd., Visiokatu 4, P.O. Box 1300, FI-33101 Tampere, Finland; tero.malm@vtt.fi; 2 VTT Technical Research Centre of Finland Ltd., Tietotie 4E, P.O Box 1000, FI-02044 Espoo, Finland; pia.willberg-keyrilainen@vtt.fi (P.W.-K.); jarmo.ropponen@vtt.fi (J.R.)

**Keywords:** cellulose, cellulose ester, foaming, foam extrusion

## Abstract

Polymer foams are widely used in several fields such as thermal insulation, acoustics, automotive, and packaging. The most widely used polymer foams are made of polyurethane, polystyrene, and polyethylene but environmental awareness is boosting interest towards alternative bio-based materials. In this study, the suitability of bio-based thermoplastic cellulose palmitate for extrusion foaming was studied. Isobutane, carbon dioxide (CO_2_), and nitrogen (N_2_) were tested as blowing agents in different concentrations. Each of them enabled cellulose palmitate foam formation. Isobutane foams exhibited the lowest density with the largest average cell size and nitrogen foams indicated most uniform cell morphology. The effect of die temperature on foamability was further studied with isobutane (3 wt%) as a blowing agent. Die temperature had a relatively low impact on foam density and the differences were mainly encountered with regard to surface quality and cell size distribution. This study demonstrates that cellulose palmitate can be foamed but to produce foams with greater quality, the material homogeneity needs to be improved and researched further.

## 1. Introduction

Polymer foams, also known as cellular plastics, are an important polymeric material. Cellular structure is formed from a combination of gaseous and solid phases. Foams can be closed-cell or open-cell in structure. In general, closed-cell foams are more rigid, whereas open-cell foams are usually flexible. Depending on the structure and material, polymer foams can have a low density, good thermal and sound insulation properties, and high specific strength. Therefore, these foam materials have been widely used in the fields of thermal insulation, acoustics, automotive, packaging, sports equipment, and construction [1,2,3,4]. Additionally, foaming is an efficient way to reduce the required amount of material and therefore cost [5].

Extrusion foaming is one of the primary foaming processes due to its continuous nature especially for polyolefins, polystyrene, and vinyl plastics [3]. In extrusion foaming, porous structures are achieved by incorporating a blowing agent into the polymer melt. Blowing agents can be divided into physical and chemical blowing agents depending on the gas production method. Physical blowing agents are, for example, low boiling point organic hydrocarbons (e.g., isobutane and pentane) [6,7,8] or inorganic materials (e.g., nitrogen, carbon dioxide, water, and argon) [7,8,9,10,11,12,13] that are added to the polymer matrix as liquid or gas and usually go through a phase change during foaming. Chemical blowing agents (e.g., sodium bicarbonate, citric acid, and azodicarbonamide) [14,15,16] produce gaseous products through chemical reactions, usually decomposition upon heating. Nucleating agents (e.g., talc and calcium carbonate) are used for controlling the cellular structure of foams (e.g., distribution, number, and size of cells). Without a nucleating agent, the number of cells might be low and the average cell size may be large, causing difficulty to produce low-density foams with uniform cell morphology [17,18].

The most widely used polymer foams are made of polyurethane (PU), polystyrene (PS), polyethylene (PE), or poly(vinyl chloride) (PVC). These conventional polymers are petrochemical derivatives and are not bio-based. However, environmental awareness, new legislative initiatives, and depletion of non-renewable resources have been boosting interest towards more sustainable bio-based materials. Considerable efforts are therefore being made to replace petrochemical-based polymeric foams with foams based on renewable resources such as starch [19,20,21,22,23] or poly(lactic acid) (PLA) [7,24,25,26,27].

Cellulose-based thermoplastics are also a promising biopolymer group for replacing synthetic foams in certain foam applications. However, only some results of cellulose-based extrusion foams have been reported [28,29,30,31,32,33,34]. Cellulose acetate (CA) is one of the cellulose derivatives that have been recognized as suitable for foam extrusion [28,30,32]. Pure cellulose acetate is thermoplastic material with a glass transition close to its thermal decomposition temperature that narrows its processing temperature area. Therefore, pure CA cannot be processed using extrusion foaming without external plasticizers [28,35]. In our previous studies, we have developed thermoplastic long-chain cellulose esters that can be processed without the addition of plasticizers. These long-chain cellulose esters are 100% bio-based material and processable at low temperatures (~130 °C) [36,37,38].

In this study, the suitability of cellulose palmitate ester for extrusion foaming was examined. The cellulose palmitate was prepared according to our earlier study. It has also been reported that the cellulose palmitate can be used to compensate for the use of fossil-based injection molding plastics and melt-spun fibers; thus, it was assumed that cellulose palmitate would also work well in the extrusion foaming process [39,40]. Foamability of cellulose palmitate was studied using three physical blowing agents. Additionally, the effect of foaming temperature on foamability was studied.

## 2. Materials and Methods

### 2.1. Materials

Commercial softwood dissolving-grade pulp purchased from Domsjö Fabriker AB (Örnsköldsvik, Sweden) was used as a starting material and the pulp was pre-treated with ozone according to the method described by Willberg-Keyriläinen et al. [36]. Lithium chloride (LiCl), N,N-Dimethylacetamide (DMAc), palmitoyl chloride, pyridine, and acetone were analytical grade and purchased from Sigma-Aldrich (Espoo, Finland). Technical ethanol was purchased from Altia Oyj (Helsinki, Finland).

Three different physical blowing agents were used to prepare the cellulose palmitate foams. Carbon dioxide, nitrogen, and isobutane R600a (IB) were purchased from Linde Group (Espoo, Finland). The blowing agent content was varied during foaming to find the optimal dosing. Finntalc M05SL (median particle size 2.2 µm, Mondo Minerals Oy, Outokumpu, Finland) was used as a nucleating agent.

### 2.2. Preparation of Cellulose Palmitate

The cellulose palmitate was prepared using the homogeneous method presented by Willberg-Keyriläinen et al. [36,37]. In this method, dry cellulose was first dissolved in a 5% LiCl/DMAc solution. Then, palmitoyl chloride 3 equivalents to the cellulose anhydroglucose unit (AGU) was added to the cellulose mixture using pyridine (3.6 equivalents/AGU) as a catalyst. The reaction temperature was 80 °C and the reaction time was 16 h. The product was precipitated with ethanol and washed first with ethanol and then acetone. The degree of substitution (DS) of the prepared cellulose palmitate was 1.0 (according to the solid-state NMR).

### 2.3. Extrusion Foaming

Foams were prepared with the Brabender Plastograph EC plus 19 mm singe screw extruder (Brabender GmbH & Co KG, Duisburg, Germany). The extruder was also equipped with a 0.66 cm^3^/rev melt pump and static mixer-type melt cooler with oil tempering. The extruder screw geometry appropriate for foaming was used that has a three-phase compression process and an increase in inner diameter at the blowing agent injection point. The extruder barrel was heated with 3 heating bands (zones) and the blowing agent was injected between zones 2 and 3. The extruder was also equipped with a round capillary die. The die geometry was 2/20 (diameter/length in mm).

Blowing agents were injected and pressurized with the Teledyne ISCO dual-syringe pump system (Teledyne ISCO Inc., Lincoln, NE, USA). As carbon dioxide and isobutane can be liquefied, they were also dosed with the Teledyne pump. The injection pressure for carbon dioxide and isobutane was 120 bar and 85 bar, respectively. In order to aid liquefaction, the pump was cooled to 2 °C. As nitrogen cannot be liquefied easily, it was injected as gas. Nitrogen dosing was conducted using the Bronkhorst EL-FLOW mass flow controller (Bronkhorst High-Tech B.V., Ruurlo, The Netherlands). Still, the Teledyne pump was used to pressurize the nitrogen gas to 200 bar. The blowing agent dosage was calculated as a mass percentage based on the material throughput determined by measuring a 1 min sample by hand. 

**Figure 1:**
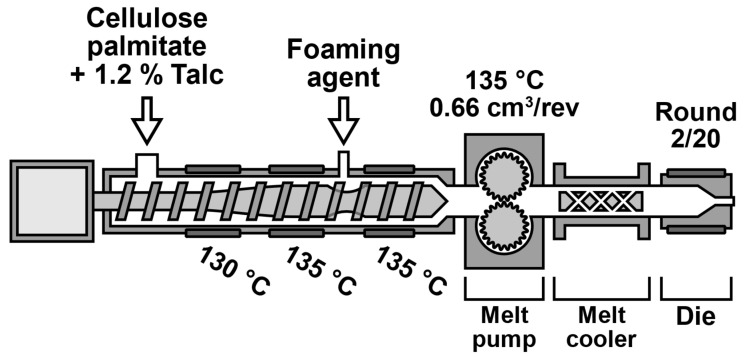
Extrusion foaming setup.

During the foam processing, the extruder barrel temperatures and melt pump temperatures were kept constant. The temperature profile was 130 °C, 135 °C, 135 °C, 135 °C. Therefore, to prepare the sample, only the melt cooler and die temperatures were changed. Other processing-related variables included the blowing agent concentration, melt pump RPM (material throughput), and die geometry. When a foam sample was taken, the die pressure was recorded with Brabender WinEXT software (Version 4.9.0, Brabender GmbH & Co KG, Duisburg, Germany). The extrusion foaming setup is presented in Figure 1.

Cellulose palmitate material was dried under vacuum at 30 °C overnight. Additionally, the extruder hopper was shielded with nitrogen gas flow (~5 L/min) to prevent the material from absorbing moisture. The nucleating agent was dry-blended by hand in a bag with the cellulose palmitate before processing. The amount of nucleating agent was kept constant at 1.2 wt%.

### 2.4. Density Measurement

Due to the irregular shapes of the prepared foams, the densities were determined with a liquid submersion technique. Water purified with reverse osmosis was used as the liquid media. Surfactant was used to reduce the surface tension of the water to prevent microbubbles from forming on the foam surfaces during submersion. Additionally, as the sample foams are closed cell and the material is impermeable to water, ingress of water into the foam cells is not expected. Four parallel measurements were made from each foam sample. The foams were weighed with the Mettler Toledo XS205 DualRange scale (Mettler Toledo, Columbus, OH, USA).

### 2.5. Scanning Electron Microscopy

Scanning electron microscope (SEM) images were taken in order to determine the cell size, wall thickness, and general cell structure. Images were captured with JEOL JSM-6360LV (JEOL Ltd., Tokyo, Japan). A small section of the foam extrudate was frozen in liquid nitrogen and fractured. The fractured surfaces were then gold-coated with the Bal-Tec SCD050 (BalTec, Balzers, Liechtenstein) sputter coater. An acceleration voltage of 7 kV was used and the images were constructed from secondary electrons.

### 2.6. Tomography

Cell size distribution was analyzed using X-ray micro-computed tomography (XµCT). Samples were imaged using the Rx Solutions desktom 130 scanner (RX solutions, Chavanod, France). An acceleration voltage of 40 kV and a voxel size of 3.7 µm were used. Scanning time was around 60 min per sample. To measure pore sizes, local thickness transform was used [41]. The local thickness transform fits spheres into the structure such that the spheres fill the void space optimally. The method defines for each voxel the maximal sphere it belongs to. This allows for the calculation of the volume-normalized probability distribution for the pore sizes.

## 3. Results and Discussion

### 3.1. Effect of Blowing Agents

The foaming parameters used in the preparation of cellulose palmitate foam samples with isobutane, carbon dioxide, and nitrogen are presented in Table 1. Samples were prepared with a melt cooler at a lower temperature than the die. The goal was to reduce the material temperature significantly while heating only the surface of the material at the die to prevent the die from clogging. The aim was also to maintain the same temperatures with varying blowing agent concentration. The sample CO_2_-0.5 was produced at a higher die temperature (120 °C) than the other CO_2_ samples (115 °C) as temperatures below 120 °C caused the material to freeze at the die. Carbon dioxide at 0.5% did not provide enough plasticization to the material in order to reach the lower processing temperature. Maximum blowing agent concentrations for isobutane, carbon dioxide, and nitrogen were 5%, 3%, and 1%, respectively. The maximum blowing agent amount was determined in-situ by evaluating the foaming behavior of the extruded material. With an excessively high amount of blowing agent, the foam would rupture prematurely, producing a slight popping sound, and collapse. The amount of maximum blowing agent is related to the blowing agent solubility to the cellulose palmitate as well as to the relationship between the material melt strength and specific volume of the blowing agent [42].

A blowing agent is known to reduce the polymer material viscosity. During the foaming process, blowing agents act as a plasticizer and increase the free volume [43,44]. To completely dissolve the blowing agent, adequate pressure is needed. To achieve adequate pressure, a certain viscosity of the polymer melt is required. This is usually achieved by reducing the processing temperature to compensate for the viscosity reduction caused by a blowing agent [28,44]. The effect can also be observed in our experiments as a reduction in die pressure when the amount of blowing agent was increased. While it is challenging to evaluate the plasticization effect of blowing agents on a material without proper measurement, die pressure can be used to determine an estimate on the matter, especially considering throughput and die geometry were kept constant. If we compare samples IB-3 and CO_2_-3 that were made with the same amount of blowing agent, they both produced a die pressure of 101 bar. However, CO_2_-3 did so with 10 °C lower melt cooler and die temperatures, indicating that the plasticization effect of carbon dioxide is higher than that of isobutane. The effect of nitrogen is more difficult to evaluate and compare due to the increased throughput. Nitrogen was used at a higher material throughput in order to increase nitrogen feeding accuracy with the utilized mass flow controller. However, generally nitrogen is far less soluble and therefore has less plasticization capability than carbon dioxide [45,46].

**Figure 2:**
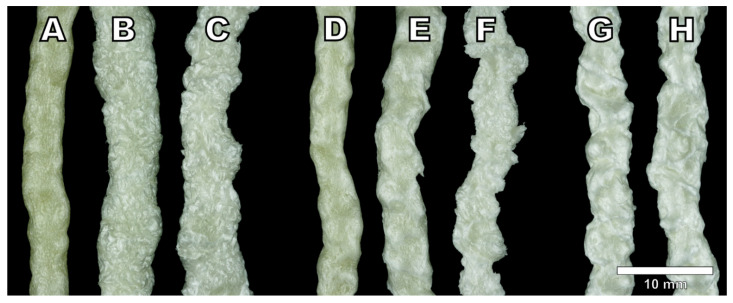
Side profiles of produced cellulose palmitate foams: (**A**) IB-1, (**B**) IB-3, (**C**) IB-5, (**D**) CO_2_-0.5, (**E**) CO_2_-1, (**F**) CO_2_-3, (**G**) N_2_-0,5, and (**H**) N_2_-1. The scale is approximate.

All three physical blowing agents were able to foam the cellulose palmitate material successfully. The visual appearance of the produced foam strands is presented in Figure 2. While foams produced with low blowing agent concentrations share some visual characteristics, foams produced with a higher concentration exhibit differences between blowing agents more effectively. Foams produced with isobutane have a higher expansion than nitrogen and carbon dioxide which are closer to each other. Surface quality or foam uniformity was not particularly good with any of the foamed samples which is probably related to the material properties themselves rather than strictly the blowing agent. However, differences in the surface quality between blowing agents can be detected. Arguably, carbon dioxide produced the most non-uniform surface for the foams at the 3% dosage. While isobutane also produced a foam with relatively rough surface quality, the surface texture was more consistent than the carbon dioxide foam. Additionally, a sample foamed with 3% isobutane had a smoother surface than the 5% isobutane sample that had clearly shrunk. Foams produced with nitrogen had the smoothest surface (looking past material irregularities) and the highest shine.

**Figure 3:**
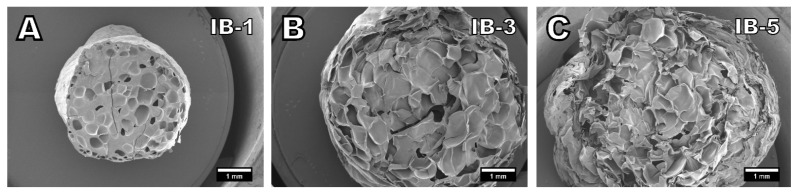
SEM images of samples foamed with isobutane in amounts of (**A**) 1%, (**B**) 3%, and (**C**) 5%. (15× magnification).

**Figure 4:**
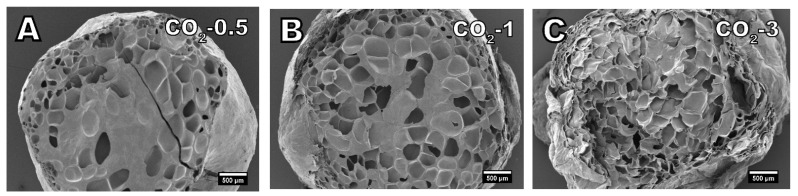
SEM images of samples foamed with carbon dioxide in amounts of (**A**) 0.5%, (**B**) 1%, and (**C**) 3%. (25× magnification).

**Figure 5:**
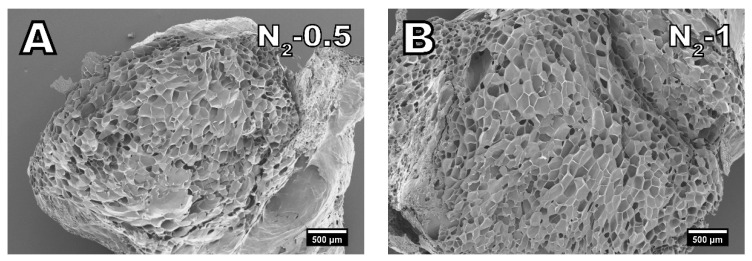
SEM images of samples foamed with nitrogen in amounts of (**A**) 0.5% and (**B**) 1%. (25× magnification).

Foam morphologies determined with SEM imaging are presented in Figure 3, Figure 4 and Figure 5. As observed by the visual appearance, the lowest amounts of isobutane (1%, Figure 3A) and carbon dioxide (0.5%, Figure 4A) produced very similar foam morphologies. The amount of blowing agent in these samples has not been high enough to fully foam the material. The formed cells are scattered around the material without neighboring cells sharing a cell wall producing a very low foaming degree. The foam densities presented in Table 2 display that the samples IB-1 and CO_2_-0.5 also have very similar densities of 0.47 g cm^−3^ and 0.45 g cm^−3^, respectively.

Samples with 3% isobutane (Figure 3B) and 1% carbon dioxide are relatively comparable in terms of densities as well. IB-3 had a density of 0.20 g cm^−3^ and CO_2_-1 had a density of 0.24 g cm^−3^. Figure 4B shows that 1% of carbon dioxide has not fully foamed the material as there are areas with no cells present, resulting in the slightly higher density. However, the cells that have formed are relatively spherical in shape and the cell structure shows no signs of collapse. The same cannot be said about the sample of IB-3 foamed with 3% isobutane. Figure 3B shows that while the material has been fully foamed, the cell structure had collapsed and the cells lost their spherical form. The cell collapse is also the reason for the rough surface quality of the IB-3 sample.

Cell collapse is more pronounced on the samples IB-5 and CO_2_-3. In particular, the sample foamed with 5% isobutane (Figure 3C) had severely collapsed cell structure to the point where it is difficult to distinguish between individual cells. This is also clearly visible based on the visual appearance of the foams (Figure 2) as IB-5 had shrunk much more than IB-3 and had a worse surface quality. Die pressure on the sample IB-5 was only 67 bar, indicating that there is still more headroom in the die pressure and the viscosity has been relatively low. Therefore, it is possible that more stable foam with less cell collapse could have been achieved with a lower foaming temperature and increased viscosity. Nevertheless, the highest expansion ratio and the lowest density was achieved with 5% isobutane. A similar trend can be seen with a sample of CO_2_-3 that had the highest amount of carbon dioxide. The cells have lost their spherical shape and the cells walls are wrinkled. This also results in rough surface quality. While carbon dioxide could benefit from reduced foaming temperature to achieve cells with better form, the effect might be more limited with carbon dioxide than isobutane due to blowing agent diffusion during aging.

When a foam structure is closed cell as these samples are, the blowing agent has to permeate through the cell walls. Simultaneously, air (mostly nitrogen) permeates into the cells, replacing the blowing agent until equilibrium is reached. Isobutane and carbon dioxide are eventually replaced by air. If the blowing agent diffusion rate is faster than that of air, the cells have a small vacuum that can cause the cells to shrink or collapse due to poor cell wall stiffness [47]; this is especially the case with carbon dioxide. Carbon dioxide is a much smaller molecule than, for example, isobutane and it tends to permeate the cells faster. This effect has been encountered with PLA foams [7]. While gas permeation values of cellulose palmitate are not known, in some cellulose based thermoplastics, permeation of carbon dioxide is higher than that of nitrogen [48].

When nitrogen is used as a blowing agent, the diffusion of nitrogen out of the cells can be considered to be in equilibrium with air diffusing into the cells. Therefore, shrinkage with nitrogen is negligible. Figure 5 displays the cell morphology of samples foamed with nitrogen. The cells in the samples N_2_-0.5 and N_2_-1 show no signs of shrinking or collapse as the cell walls are not creased. Furthermore, compared to isobutane and carbon dioxide, the material is fully foamed even at a low nitrogen dosage of 0.5%. Despite the comparatively low dosage of nitrogen, samples N_2_-0.5 and N_2_-1 have a similarly low density of around 0.19 g cm^−3^ as IB-5 and CO_2_-3. Nitrogen has the highest specific volume followed by carbon dioxide and isobutane [49]. Therefore, less nitrogen is necessary to achieve the same expansion than for carbon dioxide or isobutane.

The average cell size and cell density are a combination of several foaming parameters such as pressure drop rate, level of supersaturation, use of nucleating agents, temperature, and blowing agent [50,51]. The average cell size and cell density of the cellulose palmitate foams is different with all three blowing agents. While it is difficult to quantitatively measure the average cell size due to collapsed cell structures of carbon dioxide and isobutane foams, some visual estimations can be made. By far the smallest and most uniform cell size with the highest cell density was achieved with nitrogen. Nitrogen also produced the thinnest cell walls. Isobutane produced the largest cell size, whereas carbon dioxide produced cells between isobutane and carbon dioxide. However, considering that the pressure drop rate has a significant effect on cell density and the samples were not produced with equal pressure drop rates, the evaluation of the blowing agent effect on cell density and average cell size is slightly limited [52,53]. Nevertheless, nitrogen is known to have a better nucleation capability and to produce higher cell density than carbon dioxide [45,54]. Cell density was also increased when the blowing agent content was increased. This is expected due to the higher level of supersaturation increasing the driving force of nucleation. Similar results as discussed here were reported by Kim et al. [8] with nitrogen, carbon dioxide, and n-butane.

The density results in Table 2 indicate that the density of the cellulose palmitate foams decreased significantly as the blowing agent content increased except for samples in which nitrogen was used. In these foams, the density remained the same even though the amount of blowing agent doubled. While the solubility of nitrogen to cellulose palmitate is not known, it is possible that the additional amount of nitrogen after 0.5% has not completely dissolved in the melt. It has been reported that increasing nitrogen can have diminishing returns on void fraction and cell density in high-density foams [5,9,55].

### 3.2. Effect of Foaming Temperature

Based on the results of the blowing agent survey, one blowing agent was selected for further foaming temperature studies with cellulose palmitate. Based on cell morphology and density measurements, nitrogen would seem like the logical choice considering it produced foams with the most uniform cell structure and low density. However, while proper mechanical tests were not conducted, the strength of each foam was estimated by hand. The foams prepared with nitrogen had virtually no strength and were quite brittle in nature. Therefore, the selection of the blowing agent was narrowed down to carbon dioxide and isobutane. From these two very similarly behaving blowing agents, better surface quality and visual uniformity was achieved with isobutane. Isobutane foams exhibited significant cell collapse but based on the blowing agent diffusion rates, isobutane could benefit more from temperature adjustment or at least the effect of foaming temperature may be clearer. Therefore, isobutane was selected.

Foaming parameters used in the isobutane temperature survey are presented in Table 3. The amount of isobutane was fixed to 3%. Considering the foam prepared with isobutane had the largest average cell size, the throughput was also doubled in hopes of faster pressure drop rate, increased nucleation, and smaller average cell size. For simplicity, melt cooler and die temperature were always set to the same temperature. The foaming temperature was between 105 °C and 130 °C with 5 °C increments. The lower limit for foaming temperature was determined to be when die pressure reached 200 bar.

**Figure 6:**
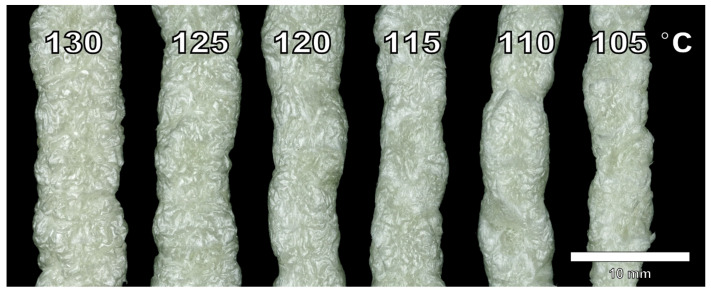
Side profiles from the produced cellulose palmitate foams using different foaming temperatures and 3% IB as a blowing agent. Refer to Table 3. The scale is approximate.

Foam side profiles presented in Figure 6 show that the surface is coarse at temperatures of 130 °C and 125 °C. When temperature is decreased, the foam surface becomes smoother. Reduction in foaming temperature also reduces the strand diameter. However, this is difficult to estimate due to the irregular surfaces. This is expected considering at a high temperature, foam expansion is governed by gas loss, and at a lower temperature foam expansion can be reduced due to an increase in viscosity and material solidification [56]. At a high temperature, the diffusion of isobutane is fast and the stiffness of the material is too low to accommodate the loss of gas. This results in high expansion but also a high amount of shrinking. At a low temperature, the blowing agent does not have enough expansion power to fully foam the material. Some melt fracture is also present at 105 °C, indicating that the lower processing temperature had been reached.

**Figure 7:**
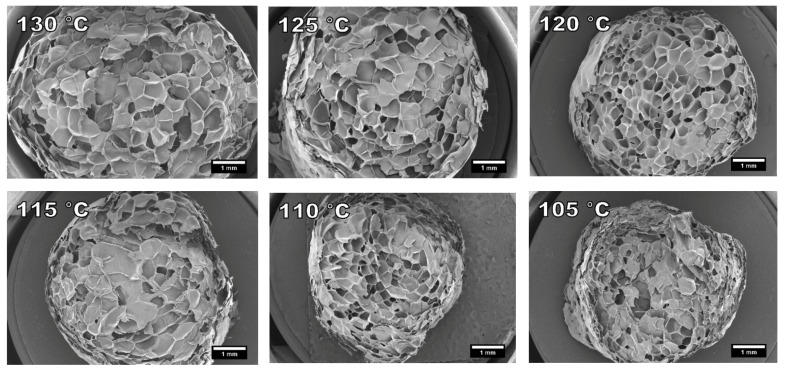
SEM images of prepared cellulose palmitate foams using different foaming temperatures and 3% IB as a blowing agent. Refer to Table 3. (15× magnification).

The cause for the shrunken surface of samples foamed at 130 °C and 125 °C is visible on the SEM images presented in Figure 7. The cells of these two samples have lost their spherical shape and the cell walls are slightly bent. Under 120 °C, the foam morphology starts to become more uniform. Based on the SEM analysis, the most uniform cell structure was achieved at 120 °C. However, the foams are not very uniform and the strands have areas of well foamed and poorly foamed material. This non-uniformity in foaming and strand diameter was increased below 120 °C. This was also apparent in the SEM analysis as the poorly foamed areas were more likely to exist in the SEM cross sections.

**Figure 8:**
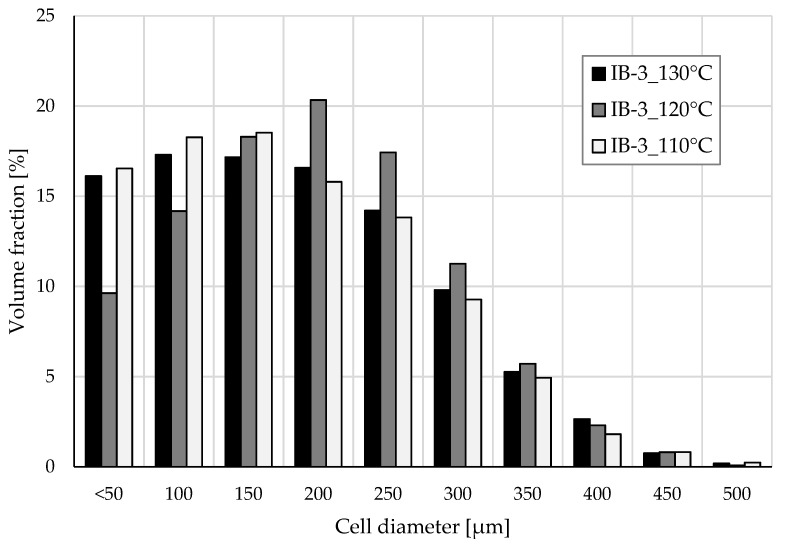
Cell size distribution of selected samples determined with XµCT.

Cell size distribution of samples IB-3_130°C, IB-3_120°C, and IB-3_110°C were analysed using tomography. The results are presented in Figure 8. Samples foamed at 130 °C and 110 °C show remarkably similar distribution in cell size despite differences in SEM analysis. Both samples have a strong presence of small cells (≤150 µm) but for different reasons. The average cell size is determined by fitting a sphere inside a measured void. Considering the foaming temperature had essentially been too high for IB-3_130°C and the cells had shrunk or collapsed slightly, the average cell size was also reduced. At 110 °C, the temperature starts to become too low and the material viscosity becomes excessively high. Therefore, it is more difficult for the isobutane to form a large cell into the material. Additionally, at a low temperature, the die pressure is increased which promotes higher cell density and smaller cells. The mean cell size for IB-3_130°C and IB-3_110°C is 182 µm and 178 µm, respectively, with similar distribution. IB-3_130°C has slightly more cells above 150 µm and especially above 300 µm that can indicate the occurrence of cell coalescence. The well-defined cell structure of Sample IB-3_120°C is also visible in the tomography data. While the average cell size achieved at 120 °C is slightly higher at 198 µm, the cell size distribution is also the narrowest. At 120 °C, the material had a suitable amount of stiffness to stabilize the cell structure, preventing shrinkage while having low enough viscosity for achieving maximal cell expansion.

Foam appearance, SEM images, and tomography data all show differences between the foaming temperatures. However, densities of the foams are surprisingly similar as evident in Table 4. While the differences are small, some conclusions can be established that support previous findings. At the low temperature of 105 °C, the density begins to increase because the foam expansion is restricted due to material solidification. Nevertheless, at the selected temperature range, foaming temperature had little effect on foam density. This is not very common as generally foaming temperature influences density [10,57,58,59,60]. Additionally, uniform cell structure can sometimes be achieved at the expense of increased foam density [10]. In the case of cellulose palmitate, relatively uniform cell structure can be achieved with isobutane without sacrificing density.

## 4. Conclusions

The foamability of cellulose palmitate was tested with three physical blowing agents: isobutane, nitrogen, and carbon dioxide. All three blowing agents were able to foam the material successfully and achieve densities below 0.20 g cm^−3^. However, major differences in foam morphology were noticed between the blowing agents. Nitrogen produced foams with the most uniform cell structure and the smallest average cell size without visible shrinking. Nitrogen was also able to foam the material with a very low dosage of 0.5 wt%. Isobutane produced foams with the largest average cell size and with visible shrinking. Carbon dioxide produced foam with an average cell size between that of nitrogen and isobutane but also with significant shrinking and poor surface quality. A higher amount of isobutane and carbon dioxide than nitrogen was required to foam the material fully.

While the average cell size with isobutane was the largest of the three blowing agents, it was still deemed to be the most suitable blowing agent to conduct foaming temperature optimization studies. Foaming temperatures in the range of 105–130 °C with 3 wt% isobutane were tested. While higher radial foam expansions were encountered more often at high temperatures (130 °C) than low temperatures (110–105 °C), foaming temperature had very little effect on foam density. Additionally, cell size distribution developed at high and low temperatures was relatively similar but for different reasons. At high temperatures, foam shrank due to poor cell wall stiffness and rapid gas loss, resulting in a higher number of small cells. At low temperatures, a higher number of small cells were developed due to the high material viscosity and increased die pressure that promotes nucleation. The optimal foaming temperature for cellulose palmitate material with 3 wt% isobutane was around 120 °C because at that temperature, the most uniform cell size distribution with low 0.19 g cm^−3^ density was achieved.

Used cellulose palmitate material was rather non-uniform which had an influence on the overall foam quality. The material uniformity has also undoubtedly masked some of the foaming characteristics caused by different blowing agents and foaming temperatures. Nevertheless, clear conclusions were able to be established. Further studies should focus on improving material uniformity for foaming purposes and greater foaming characteristics.

## Figures and Tables

**Table 1:** Foaming parameters of prepared cellulose palmitate foam samples.

Sample ID	Blowing Agent		Temperature [°C]		Die Geom.	Die Pressure [bar]	Throughput [g/min]
	Type	wt%	Melt Cooler	Die			
IB-1	Isobutane	1	120	125	2/20	125	14
IB-3	Isobutane	3	120	125	2/20	101	14
IB-5	Isobutane	5	120	125	2/20	67	14
CO_2_-0.5	Carbon dioxide	0.5	110	120	2/20	150	14
CO_2_-1	Carbon dioxide	1	110	115	2/20	163	14
CO_2_-3	Carbon dioxide	3	110	115	2/20	101	14
N_2_-0.5	Nitrogen	0.5	110	120	2/20	151	28
N_2_-1	Nitrogen	1	110	120	2/20	145	28

**Table 2:** Densities of prepared cellulose palmitate foams.

Sample ID	Density [g cm^−3^]
IB-1	0.47 ± 0.07
IB-3	0.20 ± 0.02
IB-5	0.17 ± 0.01
CO_2_-0.5	0.45 ± 0.02
CO_2_-1	0.24 ± 0.01
CO_2_-3	0.19 ± 0.01
N_2_-0.5	0.18 ± 0.00
N_2_-1	0.19 ± 0.00

**Table 3:** Processing parameters used during production of temperature survey samples.

Sample ID	Blowing Agent		Temperature [°C]		Die	Die Pressure	Throughput
	Type	wt%	Melt Cooler	Die	geom.	[bar]	[g min^−1^]
IB-3_130°C	Isobutane	3	130	130	2/20	81	28
IB-3_125°C	Isobutane	3	125	125	2/20	102	28
IB-3_120°C	Isobutane	3	120	120	2/20	130	28
IB-3_115°C	Isobutane	3	115	115	2/20	143	28
IB-3_110°C	Isobutane	3	110	110	2/20	165	28
IB-3_105°C	Isobutane	3	105	105	2/20	200	28

**Table 4:** The densities of prepared cellulose palmitate foams using different foaming temperatures and 3% IB as a blowing agent.

Sample ID	Density [g/cm^3^]
IB-3_130°C	0.18 ± 0.00
IB-3_125°C	0.18 ± 0.01
IB-3_120°C	0.19 ± 0.00
IB-3_115°C	0.18 ± 0.01
IB-3_110°C	0.19 ± 0.01
IB-3_105°C	0.21 ± 0.01
